# Support after return to alcohol use: a mixed-methods study on how abstinence motivation and app use change after return to alcohol use in an app-based aftercare intervention for individuals with alcohol use disorder

**DOI:** 10.1186/s13722-024-00457-7

**Published:** 2024-05-06

**Authors:** Catharina Lang, Kiona K. Weisel, Sebastian Saur, Lukas M. Fuhrmann, Antonie Schoenleber, Daniela Reichl, Niklas Enewoldsen, Sabine Steins-Loeber, Matthias Berking

**Affiliations:** 1 Department of Clinical Psychology and Psychotherapy, Institute of Psychology, University of Erlangen-Nuremberg, Nägelsbachstraße 25a, 91052 Erlangen, Germany; 2 Department of Clinical Psychology and Psychotherapy, Institute of Psychology, Otto Friedrich University of Bamberg, Markusplatz 3, 96047 Bamberg, Germany

**Keywords:** Alcohol Use Disorder, App, Mixed methods, Qualitative interviews, Telephone coaching, Intervention, Relapse prevention, Abstinence motivation

## Abstract

**Background:**

As the return to alcohol use in individuals with alcohol use disorder (AUD) is common during treatment and recovery, it is important that abstinence motivation is maintained after such critical incidences. Our study aims to explore how individuals with AUD participating in an app-based intervention with telephone coaching after inpatient treatment perceived their abstinence motivation after the return to alcohol use, whether their app use behavior was affected and to identify helpful factors to maintain abstinence motivation.

**Methods:**

Using a mixed-methods approach, ten participants from the intervention group of the randomized controlled trial *SmartAssistEntz* who returned to alcohol use and recorded this in the app *Appstinence*, a smartphone application with telephone coaching designed for individuals with AUD, were interviewed about their experiences. The interviews were recorded, transcribed and coded using qualitative content analysis. App use behavior was additionally examined by using log data.

**Results:**

Of the ten interviewees, seven reported their abstinence motivation increased after the return to alcohol use. Reasons included the reminder of negative consequences of drinking, the desire to regain control of their situation as well as the perceived support provided by the app. App data showed that app use remained stable after the return to alcohol use with an average of 58.70 days of active app use (*SD* = 25.96, *Mdn* = 58.50, range = 24–96, *IQR* = 44.25) after the return to alcohol use which was also indicated by the participants’ reported use behavior.

**Conclusions:**

The findings of the study tentatively suggest that the app can provide support to individuals after the return to alcohol use to maintain and increase motivation after the incidence. Future research should (1) focus on specifically enhancing identification of high risk situations and reach during such critical incidences, (2) actively integrate the experience of the return to alcohol use into app-based interventions to better support individuals in achieving their personal AUD behavior change goals, and (3) investigate what type of support individuals might need who drop out of the study and intervention and discontinue app use altogether.

**Trial registration:**

The primary evaluation study is registered in the German Clinical Trials Register (DRKS, registration number DRKS00017700) and received approval of the ethical committee of the Friedrich-Alexander University Erlangen-Nuremberg (193_19 B).

**Supplementary Information:**

The online version contains supplementary material available at 10.1186/s13722-024-00457-7.

## Background

Alcohol use disorder (AUD) as defined by the Diagnostic and Statistical Manual of Mental Disorders-5 (DSM-5) ranges from mild to severe forms based on the amount of fulfilled criteria [1], is prevalent, often chronic and linked to negative physical and mental health [1–3]. Evidence-based treatments including pharmacotherapy, withdrawal management, cognitive behavioral therapy, motivational interviewing, and prevention of the return to substance use exist [4–7] and recommendation on treatment indication are compiled in national treatment guidelines [8]. Despite the existence of various treatment forms there is room for improvement in the current treatment landscape reflected by high lifetime rates of the return to substance use for substance use disorders [9, 10], low treatment retention, and general treatment barriers [11–13].

One emerging field in mental health is digital self-help [14]. Digital self-help has great potential: access is low threshold; after the development, costs are linked to the upkeep, amount of guidance and support provided which makes them scalable; participation is private and flexible [15, 16]. Considering smartphone apps as a delivery format for mental health interventions, one of their main advantages is that they could provide support to individuals in times of need [17].

To date, research on internet interventions for reducing alcohol consumption shows promising results [18, 19]. A meta-analysis [20] examined the efficacy of technology-delivered, cognitive-behavioral interventions for alcohol use (“CBT Tech”; CBT: Cognitive Behavioral Therapy) including 15 randomized controlled trials (RCT) with heavy and at-risk drinking. The effect of stand-alone CBT Tech was not significant in contrast to treatment as usual (TAU), but there was a small, significant effect when compared to a minimal treatment control (*g* = 0.20, 95% CI: 0.22–0.38, *k*_*es*_ = 5). When CBT Tech was used as addition to TAU and compared to TAU only, the effect was even larger (*g* = 0.30, 95% CI: 0.10–0.50, *k*_*es*_ = 7) and remained stable over a period of 12 months. However, there have only been few RCT studies of app-based AUD interventions to date. In a pilot study by Gonzales and Dulin [21], 60 participants meeting the criteria for AUD were recruited from the general community and randomized to either the 6-week app-based intervention “Location-Based Monitoring and Intervention for Alcohol Use Disorders” (LBMI-A) or an internet-based motivational intervention for the reduction of alcohol use. Both interventions led to significant decreases in drinks per week and percent of heavy drinking days, but only the LBMI-A resulted in a significant increase in percent of days abstinent. Concerning app use, 71% of the LBMI-A users accessed all app modules although their app usage declined over the course of the intervention period. App-based interventions could also be used to complement in-person treatment. Participants of an RCT [22] who met the criteria for AUD received residential substance use treatment plus “Addiction-Comprehensive Health Enhancement Support System” (A-CHESS), a smartphone application designed to improve continuing care by offering emotional and instrumental support at any time. 80% of the A-CHESS participants were still using the app at the end of month four. The A-CHESS group (*n* = 170) reported a lower mean number of risky drinking days (1.39 vs. 2.75; *p* = .003) and they were more likely to be consistently abstinent (51.9% vs. 39.6%; *p* = .030) than participants who received only treatment as usual (*n* = 179). Overall, these findings support the idea of app-based treatments as a potential approach to help people reduce their alcohol use or achieve abstinence. Yet, little is known about how participants’ abstinence motivation – the motivation to become or remain abstinent – and their app use change after the return to alcohol use during the period of app use and what aspects help to maintain abstinence motivation.

Considering factors which might contribute to treatment success, several studies have demonstrated the importance of motivational aspects in the course of therapy [23, 24] as well as in the prevention of the return to substance use [25–27]. Motivational aspects are a determinant of long-term success of as well as engagement and retention in the treatment of substance use disorder [23, 27–32]. Motivation for change in particular was found to be a predictor of reduction in substance use [33, 34]. Stanick, Laudet and Sands [35] reported that abstinence commitment at the beginning of substance use disorder treatment increased the probability of treatment completion which in turn significantly increased the likelihood of maintaining abstinence for one year after treatment. They also found that, after controlling for substance use status, the amount of abstinence commitment at the end of the treatment was a predictor for the maintenance of abstinence in the year after substance use disorder treatment (OR = 2.27, 95% CI: 0.79–6.54). DiClemente, Doyle and Donovan [36] examined predictors of readiness to change, a construct related to substance use [37, 38], using baseline data from a study on combined interventions for alcohol dependence [39]. Results showed that abstinence self-efficacy, positive treatment outcome expectations, lower perceived level of stress, higher quality of life, female gender, higher drinking severity, older age, higher psychiatric comorbidity and greater percentage of days abstinent significantly predicted greater readiness to change drinking behavior. Concerning abstinence motivation at the end of substance use disorder treatment, the findings of another study [40] revealed perceived damage due to future substance use, abstinence self-efficacy, satisfaction with quality of life and number of network members in a recovery program as predictors of commitment to abstinence. To sum up, abstinence motivation seems to be crucial in substance use disorder treatment and there are several predictors for abstince motivation at the beginning and at the end of treatment, some of which may be targeted by interventions as distinct approach and avoidance goals. However, none of this evidence refers to the particular situation of the return to alcohol use, though this is a very common event during and after substance use disorder treatment.

Besides its associations with the above-mentioned aspects of treatment, abstinence motivation is also understood as an important determinant before and during the process of the return to substance use [41, 42]. Yet, there is a lack of research on what happens to abstinence motivation after the return to substance use. The return to substance use might be one critical tipping point at which the motivation following the occurrence might influence how individuals continue with abstinence and their treatment. Therefore, this study aims to examine abstinence motivation and app use, as a matter of behavior related to abstinence motivation, in the phase after the return to alcohol use in order to start filling this research gap. Understanding and assessing motivation is challenging as it requires insight into a person’s *attitudes, intentions, confidence and commitment, and decision-making ability* [43]. One manner to explore this is through qualitative research by conducting interviews with participants about their personal experiences in order to better understand processes in substance use disorder treatment [44, 45]. Qualitative research promotes a better understanding of the research question and gives context which would not be possible by using only quantitative measures [46]. Yet, the interpretation of qualitative data is always to a degree subjective, so it makes sense to supplement the knowledge gained from this with quantitative, i.e. objective, data. A mixed-methods approach creates a more powerful research outcome than either method could do on its own [46, 47]. For our study, this meant that we combined qualitative data on abstinence motivation and app use with quantitative app use data.

### Research questions

In this study qualitative interviews were conducted with individuals with alcohol use disorder from the intervention group (access to the *Appstinence* app and telephone coaching) of the *SmartAssistEntz *project who reported in the app to having returned to alcohol use. The aim of this mixed-methods study was to explore whether abstinence motivation and app use changed after the return to alcohol use and what aspects of the intervention and in general were perceived as supportive concerning abstinence and treatment motivation. Furthermore, we wanted to understand underlying motivational aspects and how these might affect behavior change. For this, we categorized reported factors of abstinence motivation into individual approach and avoidance goals.

## Methods

### Study design

Additional qualitative interviews were conducted with participants from the *SmartAssistEntz* project after they had completed the primary study’s observation period of six months. All interviews were conducted from April to June 2021 while the primary study was still ongoing for other participants - with the goal of gathering information for subsequent agile intervention development. They took place after the individual end of the observation period (*Mdn* = 8 weeks, range = 3–13) in order to have app usage data for the full six months observed. The primary study aims to evaluate the newly developed app-based intervention *Appstinence* for individuals with AUD after inpatient treatment in a randomized controlled trial compared to only access to treatment as usual. More information on the primary study, which was registered in the German Clinical Trials Register (DRKS, registration number DRKS00017700) and received approval of the ethical committee of the Friedrich-Alexander University Erlangen-Nuremberg (193_19 B), can be found in the published study protocol [48]. The qualitative aspect of this mixed-methods study complies with the recommendations on data collection, extraction and interpretation of [49] and follows the consolidated criteria for reporting qualitative research (COREQ) [50].

### Participants

Participants were included in the study who were in the intervention group with access to the *Appstinence *app, had surpassed the study observation time frame of six months of the primary study, reported at least once having used alcohol in the app and continued to use the app at least once after reporting of the return to alcohol use. General primary study participation criteria and detailed information on the recruitment procedure are described in the published study protocol [48]. The potential sample (*N* = 38; 25 men, 13 women) was approached by email with the request to participate in the telephone interviews about their experiences with the app. Interview participation was compensated with 15 Euros. Of the 38 approached, 27 were not interested in participating or didn’t respond to the request and 11 individuals (8 men, 3 women) between the age of 21 and 57 completed the interview. One interview was later excluded as the individual was intoxicated during the conversation.

### Intervention

The intervention consists of the *Appstinence* app with a use period of six months and weekly 30 min telecoaching - phone calls with psychotherapists - for the first six weeks after discharge of inpatient treatment. The goal of the coaching was to support individuals in finding appropriate aftercare, strengthen motivation, and help create an emergency plan. Participants were able to use a chat in the app to communicate with the coaches about app content. The app had four basic modules to complete and ten elective modules to choose from. Modules were for example “boosting motivation”, “management of risky situations”, “prevention of the return to alcohol use”, “coping with the return to alcohol use”, “relaxation” and “emotion regulation”. Content was based on a cognitive-behavioral approach including psychoeducation, exercises to support behavior change and motivation, and practical information on finding an aftercare program. The app consists of texts, videos, audio files and some tasks based on an Approach-Avoidance Bias Modification paradigm [51] in which dysfunctional attitudes about alcohol intake are to be pushed away and functional attitudes about abstinence are to be pulled towards the users via screen swiping. Gamification aspects were integrated, for example feedback and praise, progress indicators for abstinence and task completion and customization of an avatar. A special motivation area was included in which participants were asked to add their own individual motivators for abstinence in form of images, texts and audio files. There were app use reminders sent via push notification. Participants were also encouraged to use a daily abstinence and craving tracker in which they were asked to self-report whether they had been abstinent the day before and how intense their craving was on a 5-point Likert scale ranging from very weak to very strong. Additionally, there was an emergency area to deliver support during a crisis.

### Data collection and analysis

#### Qualitative interview

##### Data collection

Following an a priori formulated, semi-structured interview guide, the participants were interviewed by telephone and recorded via Internet telephone, known as *Voice over IP* using the applications Sipgate and PhonerLite. To design the interview guideline, first, a pool of potential questions was collected on the topic of the effect of a return to alcohol use on motivation and utilization of the app which led to the following sections: (1) *current treatment situation*, (2) *app use behavior*, (3) *motivation for app use and behavior change*, (4) *situation of the return to alcohol use*, (5) *effect of the return to alcohol use on motivation and app use*, (6) *general app evaluation*. Current treatment situation assessed whether the individual was currently receiving any type of support and whether the 7-day point abstinence was fulfilled. App use behavior assessed how the app was used including frequency, duration, and type of situation of use as well as the assessment of helpfulness of app reminders and the abstinence tracker. Motivation for app use and behavior change looked at the primary reason for wanting to achieve abstinence or to change drinking behavior and expectations before participating. Situation of the return to alcohol use confirmed the return to alcohol use reported in the app had happened, and it was inquired whether the app was used during or after the return to alcohol use and what other type of support was available in that time. Effect of the return to alcohol use on motivation and app use contained questions on how these aspects were influenced. General app evaluation aimed to explore whether the expectations had been met. The translated interview questions are displayed in Table 1.

**Table 1:** Interview Guide Questions

**1. Clarification of study period and current treatment situation**	
**1.1**	You participated in the study in the period from … to … The coaching went from … to … and afterwards you still had access to the “Appstinence” app - Is that correct?
**1.2**	Are you currently still participating in an aftercare treatment? (e.g. addiction counseling, self-help groups, psychotherapy)
**1.3**	Did you drink alcohol in the last 7 days?
**2. Use behavior**	
**2.1**	How often did you use “Appstinence” during the study period?
**2.2**	How long did you use the app on average?
**2.3**	In which situations did you usually use the app? (e.g. on the move, at home, at specific times)
**2.4**	How did use change after coaching?
**2.5**	To what extent did the push messages lead you to open the app?
**2.6**	How helpful has the abstinence counter / abstinence area been for you?
**3. Motivation**	
**3.1**	What expectations did you have of the app at the beginning?
**3.2**	What was the biggest motivation for you to change something about your drinking behavior?
**4. Return to alcohol use**	
**4.1**	Did you use alcohol once or more often during the study period?
**4.2**	Have you indicated the return to alcohol use in the app?
**4.3** **4.3.1**	Did you use the app during or after your return to alcohol use?
	If yes: In what way did the app help you during or after?
**4.4**	In what way did the coaching help you during your return to alcohol use?
**4.5**	What other support did you receive? (e.g. friends, colleagues, family?)
**5. Effects of the return to alcohol use**	
**5.1**	How did the return to alcohol use affect your motivation to change something about your drinking behavior in the weeks that followed?
**5.2**	How did you continue to use the app in the weeks after the return to alcohol use?
**6. App evaluation**	
**6.1**	Have your expectations for the app been met?

##### Data analysis

**Fig. 1:**
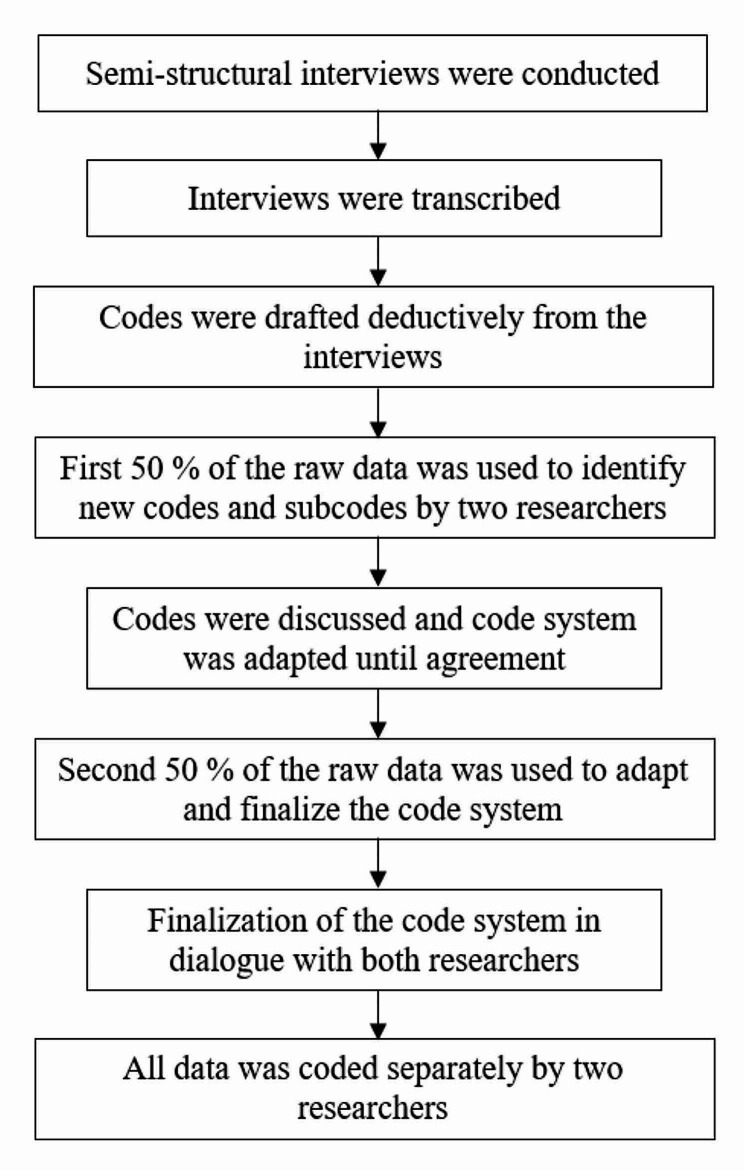
Flowchart of qualitative data analysis

The recorded interviews were transcribed using the software MAXQDA 2020 (VERBI GmbH). Transcription rules were determined prior to transcription and followed general guidelines by [52] and the analysis was based on [53]. The coding process is illustrated in Fig. 1.

The first draft of categories was deduced from the interview themes themselves, the subcodes then emerged from the data taking an inductive approach. To identify the categories, 50% of the interviews were chosen by a random generator and used to identify and create new subcodes, and adapt the already existing code system. All steps were performed by two researchers (KKW, AS). Disagreement was discussed subsequently until satisfactory agreement was reached. To validate the categorical system, the last 50% of the interview material was coded by both researchers while adapting and finalizing the code system. After this step, all material was recoded based on the finalized code system and the intercoder correlation for reliability testing was assessed. The code segment intersection rate was set to 10% as a threshold, meaning that a minimum of 10% on segment level should match with each other to count as agreement. The intercoder agreement on the final coding was 90%. The process of qualitative content analysis led to the emergence of the following categories: *7-day point abstinence, current treatment options, evaluation of app, evaluation of coaching, evaluation of inpatient detoxification, abstinence motivation, app use behavior, return to alcohol use, motivation after return to alcohol use, app use after return to alcohol use, expectations of the app, reasons to participate.*

To enhance reliability and validity six guidelines formulated by Mayring [54] were followed: (1) Documentation: Every step in the research process must be reported and justified in a comprehensible manner, (2) Validation of Interpretations: Every interpretation made must be proven and justified utilizing the available material, (3) Rule-guidedness: Data analysis must follow certain predetermined guidelines, (4) Object Proximity: Research should connect as best as possible to everyday life of the participants, (5) Communicative Validation: The codes, categories, and interpretations must be validated through an open discourse between the researchers, (6) Triangulation: Different data sources, theoretical approaches and methods should be included, i.e. by comparing qualitative and quantitative analyses.

#### Quantitative information

##### Data collection

App use data was collected and extracted from the study administration tool. All other quantitative data relevant to this study was collected in the primary study via self-report diagnostic telephone interviews and web-based assessments. For this study only descriptive outcomes for socio-demographic data and abstinence self-efficacy at baseline were included.

**Socio-demographic data** included age, gender, level of education, employment status, current occupation, location of residency, and previous diagnosis of other psychological disorders.

**Abstinence motivation and abstinence self-efficacy.** Abstinence motivation for the coming six months was assessed as well as confidence levels of achieving this abstinence on a rating scale of 1 (*not confident at all*) to 5 *(very confident*).

**App use behavior** was explored by log data and assessed by utilizing the number of days of active app use, the number of resolved tasks, the number of enters of the emergency area and the number of enters of the motivation area – in total and before and after the return to alcohol use. Alcohol use is defined here as any consumption of alcohol the previous day. Furthermore reported abstinence data was explored by using reported days of abstinence and alcohol use in the abstinence tracker.

##### Data analysis

Data is presented per person and additionally by presenting mean (*M*), standard deviation (*SD*), median (*Mdn*), range and interquartile range (*IQR*) per assessment time point.

## Results

### Study population

The total study sample was ten participants, of which seven were male, and the average age was 40.60 years (*SD* = 12.40, *Mdn* = 43.50, range = 21–57, *IQR* = 20.75). Six participants were single and four had a university degree or a higher educational level, five were unemployed at the time of the beginning of the study. Eight participants reported having at least one other diagnosed psychological disorder and five reported living in a small town or village (≤ 20.000 inhabitants). All sociodemographic characteristics are presented in Table 2. All ten participants reported to wanting to stay abstinent in the coming six months, of which five were confident and four somewhat confident to be able to reach this goal, while one person reported not being confident at all.

**Table 2:** Sociodemographic Characteristics of Participants at Time of Screening

Quantitative information					
Age (M, SD, Mdn, Range, IQR)	40.60	12.40	43.50	21–57	20.75
Male (*n*, %)	7	70			
Single (*n*, %)	6	60			
University degree or higher educational level (*n*, %)	4	40			
Currently unemployed (*n*, %)	5	50			
At least one other diagnosed psychological disorder (depression, anxiety disorder, eating disorder, personality disorder, PTSD, bipolar disorder or ADHD; *n*, %)	8	80			
Living in a small town or village (≤ 20.000 inhabitants; *n*, %)	5	50			

*Note. N* = 10

### 7-day point abstinence

Eight participants reported a 7-day point abstinence at the time of the interview, while two (P01, P09) reported to having consumed alcoholic beverages in the past seven days. The participants reported on average 117.20 days of abstinence in the abstinence tracker (*SD* = 56.05, *Mdn* = 107.50, range = 32–205, *IQR* = 78.25). These data included an initial input for days of abstinence between beginning of inpatient treatment and first app use.

### Abstinence motivation and return to alcohol use

#### Return to alcohol use

The participants reported on average 6.10 days of alcohol use in the abstinence tracker (*SD* = 4.61, *Mdn* = 6, range = 1–15, *IQR* = 6.75) over a period of six months. All interviewees were asked whether they had returned to alcohol use during the study period and whether they had reported this in the app. Although participants had been selected for this study based on recorded alcohol use in the app, two participants (P02, P10) stated to have been abstinent during the complete study period. Being asked about it, they said that they would have reported any alcohol use in the app. One participant (P07) stated to have discontinued use after the coaching period. All others reported to having used alcohol at least once and having reported this in the app. Some individuals explained their situations of the return to alcohol use, one participant (P01) stated she had returned to alcohol use due to a feeling of having been hurt by others, another (P06) stated the death of her brother had been a trigger: *“It was just a bad farewell the last four weeks and yes, bad. And then, yes, it was actually logical for me that something like that would happen”.* Others mentioned stressful situations and anger (P07), loss of contact with friends, a break-up, problems at work, and the loss of someone to talk to after the end of the withdrawal program (P09).

Five participants (P02, P04, P05, P06, P08) reported they had received support from family and friends after the return to alcohol use, three participants had received professional help from a psychologist (P06), additional counselors (P05), and a self-help group (P03). Three participants stated not having received any help, one participant felt this was due to the COVID-19-pandemic: *“[…] That was the time when everything really shut down and I had an appointment, I had already called an addiction counseling center and they said they would try me somehow, but this Corona broke so much”.* One person (P01) said she had wanted to be left alone after returning to alcohol use.

#### Abstinence motivation

Based on the answers provided, abstinence motivation was categorized in approach and avoidance goals. One major theme in the avoidance goals was fear of negative consequences. One person (P01) disclosed she had difficulty believing that she could be struggling with AUD, indicating difficulty integrating her current situation into her constructed self-image and explaining abstinence motivation. Another person (P03) expressed that she wanted to avoid social judgment by others in her self-help group as well as the study team behind the app: *“I didn’t want to have to admit to my addiction group that I had been drinking, and in there I also thought, uh, otherwise I would have to say yes, and I didn’t want to do that, so sometimes I rather didn’t drink anything because I didn’t want to admit that […] So I never knew whether someone would look at what I typed in there or not or something. And I always imagined that I would type in crap like that, maybe I didn’t disappoint them now because they don’t know me personally, but it was very uncomfortable for me to type in no”.* She was also afraid of losing her job and driver’s license. One person (P06) reported she did not want to suffer the consequences, in the short term a hangover and in the long term, weight gain.

Considering approach goals, two individuals (P10, P09) stated they wished to regain quality of life and be present in the moment: *“Yes, the recovery of my quality of life, but I really only understood that through withdrawal, then so after 3 weeks, when I then sat on a park bench, and again perceived birds chirping again, and it just got all better somehow”* (P09). The person also stated to enjoy receiving positive feedback on his appearance after a period of abstinence which additionally motivated him. There were also social aspects for abstinence motivation. One person (P07) stated he wanted to take part in social life again and three others (P09, P03, P02) mentioned their biggest motivation was their family, friends and partners. Two individuals had responsibilities towards others (P01, P06). Four individuals (P04, P05, P07, P10) reported they wanted to feel proud of themselves and their achievements and have a “*clear head*”. One person (P08) was motivated by wanting to maintain physical health and fitness and two others (P01, P02) felt they needed to stop drinking due to existing comorbid diseases.

#### Motivation after return to alcohol use

Considering change in motivation after the return to alcohol use, the following categories were determined: (1) decrease of motivation, (2) increase of motivation, (3) no change in motivation. Seven of the 10 participants reported an increase in motivation. The participants had the following explanations for their increase in motivation, two individuals (P03, P09) reported the way the app “reacted” had an impact: *“[…] and also the app, it didn’t wag its finger nastily, but you’ll manage it again tomorrow and so on. That was a motivating reaction.“; “I also thought it was good that when negative pressure was applied, that was about 5 times in the half year, that it didn’t say, oh my God, get help, but it was motivating somehow, keep going, keep at it”.* One participant (P03) said the non-judgmental support of her support group after opening up about the return to alcohol use had a positive effect. For four participants (P01, P02, P05, P07) realizing the negative effects of their drinking behavior increased their desire to regain control over their life and decisions. Two individuals (P07, P08) began to seek additional professional treatment. Three out of ten participants stated that they had an initial decrease of motivation after the return to alcohol use. They explained that they became depressed after the return to alcohol use and reported that, at first, abstinence didn’t matter anymore.

### App use

#### Quantitative app use

App use defined as days of active app use varied strongly between individuals, with a range of 31 to 181 (*M* = 109.70, *SD* = 52.42, *Mdn* = 103.00, *IQR* = 76.75). While there was no large difference in the mean number of days of active app use before (*M* = 51.00, *SD* = 36.76, *Mdn* = 41.50, range = 2–97, *IQR* = 60.00) and after (*M* = 58.70, *SD* = 25.96, *Mdn* = 58.50, range = 24–96, *IQR* = 44.25) the return to alcohol use, variance was high and a closer look at the individual app use data reveals that participants with less use days before the return to alcohol use seemed to have less use days in total. Nevertheless, both participants with an early return to alcohol use as well as participants with a later return to alcohol use used the app for several weeks after the return to alcohol use. Participants completed 86.80 tasks on average but the high range from 16 to 222 (*SD* = 59.00, *Mdn* = 63.00, *IQR* = 65.00) shows that participants differed greatly in their use behavior. The mean number of resolved tasks decreased from 55.30 before the return to alcohol use (*SD* = 36.03, *Mdn* = 49.50, range = 13–125, *IQR* = 39.75) to 31.50 after (*SD* = 53.06, *Mdn* = 13.00, range = 0-175, *IQR* = 35.00). Especially those participants with a higher number of use days before the return to alcohol use seemed to severely reduce their completion of tasks, with three of them resolving zero tasks after the return to alcohol use (P03, P04 and P09) although they still had a high number of active use days following their return to alcohol use. The number of times the emergency area was accessed ranged from 2 to 25 in total, with a mean of 10.10 (*SD* = 9.12, *Mdn* = 5.00, *IQR* = 13.25). There was no clear pattern of change in the number of access of the emergency area before and after the return to alcohol use. Participants accessed the motivation area 24 times on average, but again, the high range from 6 to 82 times of access reveals a large difference in the use behavior (*SD* = 22.34, *Mdn* = 16.50, *IQR* = 11.25). The app use data indicate a decrease in the number of access of the motivation area after the return to alcohol use, with a mean of 20.50 before (*SD* = 22.07, *Mdn* = 14.50, range = 2–79, *IQR* = 6.00) and a mean of 3.50 after (*SD* = 3.37, *Mdn* = 2.50, range = 0–11, *IQR* = 4.50) the return to alcohol use. Table 3 displays detailed information on app use of all ten participants.

**Table 3:** App use data

Participant ID	Number of days of active app use			Number of tasks completed			Number of enters of emergency area			Number of enters of motivation area		
	Total	Before return to alcohol use	After return to alcohol use	Total	Before return to alcohol use	After return to alcohol use	Total	Before return to alcohol use	After return to alcohol use	Total	Before return to alcohol use	After return to alcohol use
P01	58	34	24	123	102	21	17	5	12	26	15	11
P02	80	35	45	55	23	32	14	5	9	17	13	4
P03	158	97	61	125	125	0	3	3	0	12	11	1
P04	178	88	90	52	52	0	3	2	1	82	79	3
P05	81	25	56	222	47	175	3	0	3	16	14	2
P06	132	48	84	102	60	42	6	3	3	16	16	0
P07	31	2	29	56	19	37	2	1	1	6	5	1
P08	73	4	69	16	13	3	24	1	23	8	2	6
P09	181	85	96	70	70	0	25	17	8	19	18	1
P10	125	92	33	47	42	5	4	4	0	38	32	6
*M*	109.70	51.00	58.70	86.80	55.30	31.50	10.10	4.10	6.00	24.00	20.50	3.50
*SD*	52.42	36.76	25.96	59.00	36.03	53.06	9.12	4.84	7.29	22.34	22.07	3.37
*Mdn*	103.00	41.50	58.50	63.00	49.50	13.00	5.00	3.00	3.00	16.50	14.50	2.50
Range	31–181	2–97	24–96	16–222	13–125	0-175	2–25	0–17	0–23	6–82	2–79	0–11
*IQR*	76.75	60.00	44.25	65.00	39.75	35.00	13.25	3.50	7.75	11.25	6.00	4.50

*Note. N* = 10

#### Subjective app use behavior in general

Eight participants reported using the app on a daily basis, mainly to complete the abstinence tracker. The other two (P05, P07) reported having used the app once or twice a week. Some (P01, P02, P09) stated that their use frequency decreased over the study period of six months. Seven participants stated their average use was about ten minutes a session while three (P04, P05, P09) reported to having used the app for about one hour a session. When asked in what situations and when the interviewees used the app, five (P01, P02, P04, P05, P06) reported to having used the app when they felt they had spare time and one individual (P03) was using it every evening. One person (P08) used the app when he was feeling especially good while two others (P01, P08) used it when they felt they were struggling. Two participants (P01, P04) stated they used the app when feeling craving and one participant (P10) described use when passing through high risk situations: “[…] *that was when I was out and about, for example when I passed a beer garden, or a restaurant or so*”. Two (P08, P01) stated they used the app in situations of alcohol use. Prompted on further situations individuals used the app, one (P09) stated in situations of boredom, two (P03, P08) wanted to keep engagement in the abstinence tracker high and one person (P07) said that he used it before preparing for the coaching which was part of the intervention. He also stated to have discontinued app use after the coaching was finished.

Four individuals clearly stated that the end of the coaching sessions did not affect their use behavior. In response to whether the reminder push notifications increased use, six participants answered affirmatively, two (P09, P06) stated not having received the reminders as their app use was already on a daily basis and one (P02) did not receive reminders due to technical issues. One person (P07) stated the reminders did not motivate app use.

#### Subjective app use behavior after return to alcohol use

Seven participants (P01, P02, P03, P04, P05, P06, P08) stated their app use behavior had not changed after the return to alcohol use. Two (P02, P09) had reduced their use frequency, one person (P02) because he attended a rehabilitation program. Upon further exploration, five participants used specific content in the app after the return to alcohol use, two (P02, P03) reported frequenting the motivation section, one (P03) chose to do the “Swipe” exercises: *“I even did one of those exercises with pushing away and stuff, I thought who knows even if it doesn’t seem to make sense to my mind. But for the psyche it might be good anyway, and I just did that, just, again without much expectation, but I did it and thought, who knows, might be helpful”.* Two participants (P08, P09) mentioned having used the emergency plan and one person (P05) engaged in daily practice to be mindful of his current situation and heighten awareness for risky situations.

### Helpful and hindering factors of the intervention regarding abstinence motivation

#### Helpful and hindering factors of the app regarding abstinence motivation

Statements about the app could be divided into (1) positive and (2) negative evaluations, and (3) general suggestions for adaptions. All participants mentioned at least one positive aspect. One participant (P09) felt supported by the daily notion for reflection and confrontation with their problems. Another participant stated something similar and added that the app could not be a total treatment substitute *“[…] using the app alone is like medication, it can only be supportive, but can’t replace everything”* (P08). Two individuals (P04, P05) found the diversity of content appealing and one person (P05) liked the interactive approach. *“The interactive approach has definitely brought me a lot, so that when you read something, you tend to digress or forget that you’ve read it, but when you record it as an audio file or write it down and photograph it or whatever, then the learning material simply sticks better and the content simply sticks better”.* Three individuals (P01, P09, P10) stated that they found the section for personal motivation particularly helpful, in which they were able to upload a personal photo. One participant (P09) exclaimed that he liked that there were daily exercises he could do and highlighted the emergency plan.

The daily abstinence tracker was rated positively by all interviewees. For some it was motivating to see how many days of abstinence they had already achieved, *“I find that very helpful, I now also have that in an app that counts that, […] because that keeps the success in front of your eyes”* (P02), and the daily reminder helped: *“It helped me to then be reminded every day and I had to answer the couple of questions and yeah, I was looking forward to it part of the time that I had to do it or be reminded of it”* (P10).

Negative evaluations of the app included that one participant (P01) was displeased that abstinence days could not be reported retrospectively but had to be reported daily. One participant (P05) criticized the general structure of the app: *“Because there is no logical sequence of questions and topics, but because you have to find your own way around and then the app suggests, for example, some other module and you say, no, I don’t really want that now and I would have preferred a tighter guidance in quotation marks or a more structured guidance […]”.* Another comment was dissatisfaction with the *Swipe* exercise about feeling it was too basic: *“I kind of felt like I was in kindergarten there, I mean when I see pictures there of someone drinking alcohol and I’m supposed to press no there, I feel like I’m taking an idiot test 20 years ago, so that wasn’t that thrilling.”* Further comments were that one person (P07) would have needed more pressure to engage in the program and another (P06) stated she believed her craving would still be strong even if she used the app in personal risk situations. She also felt the app was overloaded with content thus not being able to complete all modules.

Some participants also made suggestions for improvements of the app, for example to have all days of abstinence displayed per month for an overview (P01), one participant (P09) felt he would like more support after alcohol use was reported and suggested the implementation of a peer chat group to communicate with others: *“Especially in these times when you’re not allowed to meet, when you’re there, this exchange is very important, because I’m still in contact with former drinkers who have all made it and so on, the solidarity is insane.”*

#### Helpful and hindering factors of the coaching regarding abstinence motivation

Four individuals (P03, P06, P08, P10) commented positively on the coaching while two of them (P06, P08) returned to alcohol use during the first weeks, when telecoaching was still ongoing. One person (P06) recalled the support of the coaching after her return to alcohol use: *“She also talked to me on the phone in the meantime, where I wrote that it had happened that I had been drinking. And I wrote that to her as an email and then she called me, and I thought that was totally super.”* One person (P01) who also returned to alcohol use during coaching period felt the coaching was not helpful: *“Yes, […] I got along well with the lady, but for me it’s just hard when they do not know me. To me so many things are simple, and then I have to tell her something, and then tell her something else, so that she understands the context, why and back and forth”.*

## Discussion

### Principal findings

In this mixed-methods study, we examined abstinence motivation and app use behavior after the return to alcohol use and aimed to identify the perceived supporting aspects regarding abstinence motivation. Overall, the results show an increase in abstinence motivation after the return to alcohol use and a stable app use.

Most of the participants stated that they were more motivated after the return to alcohol use. Williams and colleagues [55] found that patients with greater alcohol use show more readiness to change. Since the return to alcohol use can be seen as greater alcohol use or rather higher AUD symptom severity than before the return to alcohol use, the increase in motivation after the return to alcohol use would be an extension of the study by Williams and colleagues. Becoming aware of the negative consequences of the drinking behavior and the desire to regain control over one’s own life were the most common reported reasons for the increased motivation. The content in the app directly addresses these issues, which suggests that the app can serve as a mean to deliver motivational content. This raises the question if the app content might also be crucial in ending an ongoing return to alcohol use. Additionally, the manner in which the app was programmed to respond upon recording a return to alcohol use was described as an appreciative and encouraging reaction. Another factor was the non-judgmental support of a self-help group as aftercare. This is not surprising as respectful and supporting feedback and interactions are also part of Motivational Interviewing which has already been shown to be effective in the treatment of substance use disorder [25]. Altogether it might be that a return to alcohol use could only have a motivational impact if the return to alcohol use was adequately addressed and if affected individuals were supported in an appreciative manner. Yet, this has to be examined systematically, for example by comparing intervention and control group regarding their motivation after the return to alcohol use.

The motivation to use the app seemed to be stable after the return to alcohol use which reaffirms the findings of an increase in abstinence motivation since abstinence motivation should be crucial for an individual’s drive to continue to use the app. Although participants differed greatly in their individual app use behavior, there was no apparent change in the pattern of the app use concerning the number of days of active app use. This finding is in line with the study of Gustafson et al. [22] in which participants were still using the app A-CHESS four months after start of the intervention. Maybe the increase in motivation after the return to alcohol use has the effect of compensating an otherwise possible decline in the app use over time [21], which has yet to be confirmed by further research. All in all, one may argue that different interventions vary in their ability to motivate AUD patients, which would explain mixed results regarding intervention adherence. However, the earlier a return to alcohol use occurred, the less days of active app use were observed in total – although participants with an early return to alcohol use as well used the app for several weeks after the return to alcohol use. A plausible reason for this could be that someone who returns to alcohol use earlier might be generally less motivated and might therefore stop using the app earlier – even if there is a possible increase in motivation at first. The findings also raise the question why some people tend to stop participating in the intervention earlier than others and if an early return to alcohol use might predict an earlier discontinuation of an intervention. If an early return to alcohol use was found to be a predictor for an early intervention dropout it could be helpful to lengthen the abstinence period so that participants would use the intervention for a longer time and thus do more exercises. This should improve their self-efficacy and their AUD recovery prognosis. To achieve this, factors that are related to app use need to be identified to optimize tailoring of the intervention. Moreover, other potential predictors for an intervention discontinuation should be examined as well to facilitate the best possible adaptation of the intervention.

Most of the participants reported to having used the app daily – especially because of the abstinence tracker – and that their app use did not change after the return to alcohol use. These subjective reports about app use match up to the above-mentioned objective app data that showed no change of use after the return to alcohol use, demonstrate a stable motivation to use the app and therefore further corroborate the findings that abstinence motivation, as a driving force for stable app use, increased after the return to alcohol use. In addition, participants stated that they had used the app particularly during free time, when they experienced craving, in risk situations or when they had strong positive or negative emotions. This leads to the conclusion that the app can be a motivational support in AUD-related crisis and may possibly even prevent a return to alcohol use in those individuals who use the app in these kind of risky situations. Additionally, the varying use situations show that participants have different needs when using the app which speaks for the requirement of personalization and individualization of app-based AUD interventions.

Altogether, the app may have helped maintain abstinence motivation even after the return to alcohol use which may in turn have been a driving factor for continued stable app use after the return to alcohol use.

Considering underlying motives for abstinence, participants mentioned more approach than avoidance goals. This is consistent with the reported reasons for the increase in motivation after the return to alcohol use since the majority of these reasons (besides becoming aware of the negative consequences of drinking) could be rated as approach goals as well. The reported approach and avoidance goals, especially the fear of negative consequences of drinking and the desire to regain quality of life, also correspond to some of the aforementioned predictors of abstinence commitment at beginning and end of treatment [36, 40] which in turn was associated with the maintenance of abstinence in the year after treatment [33–35]. Prior research provided evidence that the pursuit of a high proportion of avoidance goals relative to approach goals was harmful to one’s psychological functioning and well-being [56]. Wollburg and Braukhaus [57] examined the relevance of approach and avoidance goals for treatment outcome using a sample with depressed inpatient individuals. Having just one goal phrased in avoidance terms was linked to less improvement of symptoms, though they did not hinder goal achievement. Another study on approach and avoidance goals in the prevention of a return to alcohol use with sexual offenders [58] showed that participants in an approach-focused intervention vs. an avoidance-focused intervention were more willing to report a return to alcohol use, had a higher treatment engagement and were rated by therapist to have a higher end-of-treatment motivation to live without offending. Transferring these results into substance use disorder samples, it may be important to encourage approach goals in AUD treatment and focus more on the positive consequences of behavior change and abstinence.

### Strengths and limitations

To the best of our knowledge, this is the first mixed-methods study to examine motivation after the return to alcohol use, to explore whether and how a possible change of motivation is reflected in participants’ app use behavior and to identify helpful factors for maintaining motivation. The advantages of qualitative research are manifold [e.g. 59, 60, 61]. By using a mixed-methods approach, we tried to gain a deeper understanding of the underlying factors of the examined motivational change and aimed to take the complexity of the participants’ experiences into account. We combined the qualitative information with quantitative data in order to get to a more complete picture of the investigated research questions [46, 47, 62].

Despite this mixed-methods design, there are certain limitations to our study. Our sample size was small and there was a possible selection bias since we included particularly those individuals who used the app to report their return to alcohol use and who voluntarily completed the interview. These participants might be more engaged in their treatment generally and also have higher abstinence motivation. Since this type of app use behavior would already assume some kind of motivation, it was less likely in the beginning that participants express decreased motivation after the return to alcohol use. Although there were participants that did not experience an increase in motivation after the return to alcohol use, this still remains a limitation. It could also be seen as a limitation that the study included only three women. Yet, the gender distribution is representative as it corresponds to the higher prevalence in men concerning substance use disorders in general [63] and AUD in particular [64]. Furthermore, our assessment of the explored change in motivation was retrospective, which could have influenced the findings.

### Future research

Future research should utilize the derived information of this study to improve digital interventions for AUD treatment. This could be achieved in a number of ways. First, interventions should focus more on motivation by incorporating the aspects that were perceived as supporting after the return to alcohol use. Second, these interventions should implement the app functions and factors that were evaluated as helpful by the participants, for example the abstinence tracker. Third, app factors that were evaluated as not helpful or hindering should be eliminated. For example, an algorithm for task sequence based on individual needs could fulfill the need for a tighter guidance. Fourth, the use of approach goals before and after the return to alcohol use might be of advantage, which also needs to be explored regarding AUD treatment. These adaptations may lead to more motivation and adherence and thereby drive conversion into AUD-related behavior change.

Regarding the app “Appstinence” that was used by participants in our study, future research should examine whether this app is actually able to assist AUD patients with ending a return to alcohol use or even with preventing a return to alcohol use in risk situations. These hypotheses need to be tested in an appropriate study design by implementing a quantitative approach and using an adequate sample size.

Next, future studies should include individuals who dropped out of the intervention directly after the return to alcohol use, i.e. participants who returned to alcohol use but do not report this in the app, to prevent potential selection bias. As participants with an earlier return to alcohol use stopped using the app earlier, future research should also reach out to these individuals and to individuals who dropped out of the intervention in general to find out what factors could keep them motivated and more adherent and to identify predictors of intervention discontinuation. Presumably, a better personalization of content to meet individual needs may be crucial to attain this goal.

## Conclusions

The findings suggest that abstinence motivation seems to generally increase after the return to alcohol use for participants in an app-based guided intervention for treatment of AUD. Future interventions should focus on motivation to deliver better support before and after a return to alcohol use and thereby potentially improve adherence and treatment outcomes. Furthermore, future studies need to reach out to individuals who drop out of the intervention after the return to alcohol use and to those with an early return to alcohol use.

### Electronic supplementary material

Below is the link to the electronic supplementary material.

Supplementary Material 1. An additional table shows the checklist for the consolidated criteria for reporting qualitative studies in detail [see Supplementary Table 1, Consolidated criteria for reporting qualitative studies (COREQ): 32-item checklist; “Support_after_return_to_alcohol_use_COREQ.docx”].

## Data Availability

The data generated and/or analysed during the current study are not publicly available due to privacy reasons but are available in pseudonymised form from the corresponding authors on reasonable request.

## Abbreviations

AUD: Alcohol Use Disorder
DRKS: German Registry of Clinical trials
DSM-5: Diagnostic and Statistical Manual of Mental Disorders-5
CBT: Cognitive Behavioral Therapy
RCT: Randomized controlled trial
TAU: Treatment as usual
LBMI-A: Location-Based Monitoring and Intervention system for Alcohol Use Disorders
A-CHESS: Addiction-Comprehensive Health Enhancement Support System
COREQ: Consolidated criteria for reporting qualitative research
