# Supplementary material for: Support after return to alcohol use: a mixed-methods study on how abstinence motivation and app use change after return to alcohol use in an app-based aftercare intervention for individuals with alcohol use disorder
Source: Addict Sci Clin Pract. 2024 May 6;19:35. doi: 10.1186/s13722-024-00457-7 (PMC11071226; doi:10.1186/s13722-024-00457-7)
Supplement: Supplementary file 1 — Supplementary Material 1. An additional table shows the checklist for the consolidated criteria for reporting qualitative studies in detail [see Supplementary Table 1, Consolidated criteria for reporting qualitative studies (COREQ): 32-item checklist; “Support_after_return_to_alcohol_use_COREQ.docx”]. [file 13722_2024_457_MOESM1_ESM.docx]

| **Supplementary Table 1**  Consolidated criteria for reporting qualitative studies (COREQ): 32-item checklist | | |
| --- | --- | --- |
| **No. Item** | **Guide questions/description** | **Reported** |
| **Domain 1: Research team and reﬂexivity** | | |
| *Personal Characteristics* | | |
| 1. Interviewer/ facilitator | Which author/s conducted the interview or focus group? | Schoenleber, Antonie, B. Sc. |
| 2. Credentials | What were the researcher’s credentials? E.g. PhD, MD | B. Sc. candidate |
| 3. Occupation | What was their occupation at the time of the study? | B. Sc. candidate |
| 4. Gender | Was the researcher male or female? | Female |
| 5. Experience and training | What experience or training did the researcher have? | Advanced B. Sc. study of psychology |
| *Relationship with participants* | | |
| 6. Relationship established | Was a relationship established prior to study commencement? | No |
| 7. Participant knowledge of the interviewer | What did the participants know about the researcher? e.g. personal goals, reasons for doing the research | The participants knew all tasks and responsibilities of the researcher in the project.  Personal goals: none.  Reasons for doing the interviews were known and stated before and at beginning of the interviews. |
| 8. Interviewer characteristics | What characteristics were reported about the interviewer/facilitator? e.g. Bias, assumptions, reasons and interests in the research topic | Participants were told that the interviewer is interested in the participants’ experience with the app and that she would like to write a thesis about this subject. |

| **Supplementary Table 1**  COREQ (continued) | | |
| --- | --- | --- |
| **No. Item** | **Guide questions/description** | **Reported** |
| **Domain 2: study design** | | |
| *Theoretical framework* | | |
| 9. Methodological orientation and Theory | What methodological orientation was stated to underpin the study? e.g. grounded theory, discourse analysis, ethnography, phenomenology, content analysis | Content Analysis,  Theory (TDF) basis of interview guide, inductive and deductive content analysis |
| *Participant selection* | | |
| 10. Sampling | How were participants selected? e.g. purposive, convenience, consecutive, snowball | Purposive |
| 11. Method of approach | How were participants approached? e.g. face-to-face, telephone, mail, email | Email |
| 12. Sample size | How many participants were in the study? | 10 (final sample size after exclusion of one interview) |
| 13. Non-participation | How many people refused to participate or dropped out? Reasons? | 27 out of 38 approached individuals did not want to participate; one of the 11 interviews had to be excluded due to intoxication of the interviewee |
| *Setting* |  |  |
| 14. Setting of data collection | Where was the data collected? e.g. home, clinic, workplace | Home of the participants; interviews were conducted via Phonerlite/Sipgate (Voice over IP) |
| 15. Presence of non-participants | Was anyone else present besides the participants and researchers? | No |
| 16. Description of sample | What are the important characteristics of the sample? e.g. demographic data, date | Participants of the intervention group of the primary study *SmartAssistEntz* (18 years or older, AUD diagnosis) who had surpassed the study frame, reported a relapse in the app and continued to use the app at least once after reporting the relapse (description made) |
| *Data collection* |  |  |
| 17. Interview guide | Were questions, prompts, guides provided by the authors? Was it pilot tested? | Questions, prompts & guides were provided. Pilot testing with the first interview. Reflection about items after the first interview. No difficulties to be considered. |

| **Supplementary Table 1**  COREQ (continued) | | |
| --- | --- | --- |
| **No. Item** | **Guide questions/description** | **Reported** |
| 19. Audio/visual recording | Did the research use audio or visual recording to collect the data? | Audio recording was used |
| 20. Field notes | Were ﬁeld notes made during and/or after the interview or focus group? | Yes, field notes were made during the interview |
| 21. Duration | What was the duration of the interviews or focus group? | 30 minutes |
| 23. Transcripts returned | Were transcripts returned to participants for comment and/or correction? | No |
| **Domain 3: analysis and ﬁndings** | | |
| *Data analysis* |  |  |
| 24. Number of data coders | How many data coders coded the data? | 2 |
| 25. Description of the coding tree | Did authors provide a description of the coding tree? | Yes |
| 26. Derivation of themes | Were themes identiﬁed in advance or derived from the data? | Themes identified in advance as part of the theoretical framework and derived from the data as an inductive approach as well |
| 27. Software | What software, if applicable, was used to manage the data? | MAXQDA 2020 (VERBI GmbH) |
| 28. Participant checking | Did participants provide feedback on the ﬁndings? | No |
| *Reporting* |  |  |
| 29. Quotations presented | Were participant quotations presented to illustrate the themes/ﬁndings? Was each quotation identiﬁed (e.g. ID)? | Yes |
| 30. Data and ﬁndings consistent | Was there consistency between the data presented and the ﬁndings? | Yes |
| 31. Clarity of major themes | Were major themes clearly presented in the ﬁndings? | Yes |
| 32. Clarity of minor themes | Is there a description of diverse cases or discussion of minor themes? | Yes |
| Note. Tong, A., Sainsbury, P., & Craig, J. (2007). Consolidated criteria for reporting qualitative research (COREQ): a 32-item checklist for interviews and focus groups. International journal for quality in health care, 19(6), 349-357. https://doi.org/10.1093/intqhc/mzm042 | | |
